# Practical Points

**Published:** 1905-12-16

**Authors:** 


### PRACTICAL POINTS.

### HOSPITALS, ASYLUMS, AND PAUPER BURIALS.

Dr. Jamieson B. Hurry, Abbotsbrook, Reading, has asked us to answer the following question :—

The relieving officer in a country district refuses to bury paupers who die in the hospital. In 16 & 17 Vict., § cxx., dealing with "Expenses of the burial, removal, or discharge of a pauper," it is stated : "On the death, discharge, or removal of any pauper from any asylum, registered hospital, or licensed house, the necessary expenses attending the burial, discharge, or removal of such pauper shall be borne by the union or parish (if any) to which such pauper is chargeable." What is meant here by "registered hospital"?

.˙. The foregoing quotation is from Section 120 of the Lunatic Asylums Act, 1853 (16 & 17 Vict., chap. 97), but that Act was entirely repealed and superseded by the Lunacy Act, 1890, and is therefore not now in force. "Registered hospital" in that Act meant a hospital registered under the Lunacy Acts for the reception of lunatics. Hospitals for lunatics must still be registered by the Commissioners in Lunacy, Section 231 (9), of the Lunacy Act, 1890, providing that "no lunatic shall be received in any hospital unless the same has been registered before the passing of this Act or is registered under a provisional or complete certificate by virtue of this Act." The expression "hospital" is defined by Section 341 of the 1890 Act as "any hospital or part of a hospital or other house or institution (not being an asylum) wherein lunatics are received and supported wholly or partly by voluntary contributions, or by any charitable bequest or gift, or by applying the excess of payments of some patients for or towards the support, provision, or benefit of other patients."

The payment of the expenses of burial of a pauper lunatic is met by Section 297 of the Act of 1890, which provides that "the necessary expenses attending the removal, discharge, or burial of a pauper lunatic in any institution for lunatics shall be borne by the union to which the lunatic is chargeable, or the local authority liable for his maintenance, and shall be paid by the Guardians of the union or by the treasurer of the local authority."

As regards the particular case referred to of refusal on the part of a relieving officer "to bury paupers who die in the hospital," the applicability or otherwise of the foregoing provisions of the Lunacy Act, 1890, depends, of course, upon the nature of the hospital; whether, in fact, it is a hospital for lunatics and the dead paupers are pauper lunatics.

#### The Duty of Burial.

The duty of burying a body of a pauper dying in a hospital falls upon the hospital. The Guardians or overseers cannot be compelled to throw the burden of burial upon the Poor-law, but they are empowered, if they think fit, to bury, at the expense of the poor rate, any pauper within their parish or union, whether the death takes place in a hospital or not (7 & 8 Vict., c. 101, Section 31). In practice the bodies of paupers dying in hospitals are frequently buried by the Poor-law authorities. The relieving officer, however, has no authority, without the sanction of Guardians or Overseers, to inflict the burden of a burial on the poor rate.

